# Targeting H(i)ck education for cancer therapy?

**DOI:** 10.18632/oncoscience.388

**Published:** 2017-12-28

**Authors:** Matthias Ernst, Robert J.J. O'Donoghue, Ashleigh R. Poh

**Affiliations:** Olivia Newton-John Cancer Research Institute, La Trobe University School of Cancer Medicine, Heidelberg, VIC 3084, Australia

**Keywords:** Hck, myeloid cells, macrophages, polarization, cancer

According to populist dictionaries, “Hick” describes a poorly educated person of provincial existence. As much as one can draw parallels between individual characters of *homo sapiens* and species of proteins, the cellular consequences of altering Haematopoietic Cell Kinase (HCK, pronounced “Hick”) activity are reminiscent of “Hicks”. Suspected for a while to shape the phenotype of monocytes and peripheral macrophages, HCK activity indeed regulates the evolutionarily conserved contributions of these cells to host defence, wound healing and tissue repair [1]. Recent reports suggested that HCK may also contribute to the growth and progression of malignancies and thus, by virtue of being a molecule with catalytic activity, HCK could provide a therapeutic target for small molecule anti-cancer compounds [2,3].

HCK is a myeloid and B-cell specific member of the SRC family of tyrosine kinases (SFKs) where the prototypical member c-SRC confers neoplastic transformation when constitutively activated. The latter not only occurred following the Rous sarcoma virus' hijacking of a carboxyl terminally truncated version of c-SRC or of viral proteins corrupting the functions of SFKs, but also as a consequence of SFKs acquiring carboxyl terminal non-sense mutations. Meanwhile, the cellular homologues of SFKs play important roles in signal transduction from growth factor and immune cell receptors, as well as of various immune-cell integrins. Accordingly, genetic *HCK* ablation impaired phagocytic activity of macrophages *in vitro* and LPS-induced endotoxemia *in vivo* and reduced host-defence against Listeria parasites [4]. In contrast, aberrant activation of a *de novo* oncogenic HCK fusion protein in hematopoietic cells can result in acute myeloid and other leukemias, while excessive HCK activity in the “right cells at the right time” promotes macrophage and neutrophil migration, rather than causing cellular transformation [5]. However, using such knock-in mice as hosts for solid cancer xenografts suggests that the level of HCK activity in the host is directly proportional to the capacity of primary tumors to grow and for their corresponding secondaries to thrive at metastatic sites [2]. Although this functional correlation still needs validation in suitably “humanized” animal model, elevated *HCK* gene expression or catalytic activity correlates with poorer survival for colon cancer patients.

Myeloid HCK activity promotes solid cancer growth by regulating polarization of macrophages and most likely neutrophils: the more HCK activity the stronger the expression of genes associated with alternative activation characterized by an anti-inflammatory M2 endotype [2], which supports angiogenesis and tissue repair associated with expression of scavenger receptors and the production of large quantities anti-inflammatory cytokines. Interestingly, and unlike the classical cytokine stimuli IL4/13, TLR/IL1/immune complexes and IL6/10-mediated induction of the M2a, M2b, and M2c macrophage endotypes, respectively, excessive HCK activity induces hallmarks of various shades of alternative activation. Accordingly, HCK activity in macrophages correlates with their capacity to express Arg-1 and the mannose-6-phosphate scavenger receptor CD206. Meanwhile, expression of the M2a marker TIE2 suggests that excessive HCK activity may promote tumor growth at least in part through angiogenesis, consistent with reduced CD31 expression in tumors upon genetic deletion or pharmacological inhibition of HCK [2]. Interestingly, HCK deficiency in mice has not been linked to impaired homeostasis of the continuously renewing intestinal mucosa, despite a majority of macrophages in the colon being of the M2 endotype and the findings that M2 polarization facilitates mucosal repair following experimental colitis [6]. Indeed, concurrent expression of different SFKs may provide a balance between negative and positive signals so the simultaneous ablation of HCK and LYN may promote M2 polarisation by impaired antagonistic activity against FGR [7]. However, in mast cells HCK appears to counteract LYN thereby fine tuning mast cell degranulation, which can promote angiogenesis in various models of solid tumors [8].

While adoptive transfer experiments show that HCK affects bone marrow-derived macrophages and possibly their monocytic Ly6G precursors, it remains unclear whether HCK also regulates polarization of tissue-resident macrophages derived from a MYB-positive yolk sac precursor. However our unpublished observations suggest that HCK activity also hinders elimination of (transplanted) cancer cells by the immune system. This is likely to involve the adaptive immune system as suppression of HCK appears to augment the anti-tumor immune response conferred by immune checkpoint inhibitors on immunogenic cancer cells as well.

There remains the question as to where HCK fits in to the signalling cascades that may sense a need for polarization, mediate it and ultimately execute the effect on neoplastic epithelium. HCK is physically associated with the receptors for CSF-1, the IL6-family and other cytokines implicated in regulating macrophage endotypes. Whether the physical association of HCK with leukocyte adhesion receptors may prime or reinforce macrophages for polarization by cytokines remains unknown. Strikingly, excessive HCK activity still endows an M2 endotype in the absence of STAT6 and therefore limits responsiveness to IL4/13 as the classical inducers of alternative macrophage activation [2]. But how does HCK activity in macrophages bestow growth on epithelial tumor cells? Besides the aforementioned mechanisms including angiogenesis and suppressed anti-tumor immunity, the emerging correlation between excessive myeloid HCK and elevated STAT3 activity in epithelial cancer cells is likely to be of mechanistic relevance. Elevated STAT3 not only triggers transcription of genes underpinning many of the cell intrinsic hallmark characteristics of cancer, but is also likely to confer invasive characteristics associated with a (cancer) stem cell-like phenotype. Our preliminary results suggest that the IL6-family of cytokine may serve as the link between myeloid HCK activity and excessive STAT3 activity in tumor cells.

To return to the “Hick” analogy, macrophages remain highly susceptible to the poor education signals provided by adjacent cancer cells and designed to support tumor progression through enhanced nutrient supply and establishing an immune tolerant shield. As a regulator of macrophage polarization, HCK may stand up as an alternative to PI3Kγ and CSF-1R, which are pursued as clinical targets for re-educating tumorigenic M2 macrophages to adopt a tumoricidal M1 endotype. Whether medicinal chemistry can overcome the challenges of identifying small molecules inhibitors with sufficient specificity to overcome the extensive molecular similarity within the 10 members of the SFK remains to be seen.

**Figure 1:**
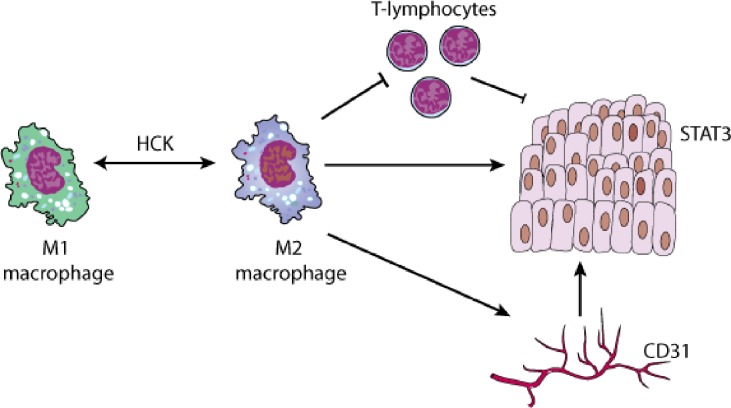
HCK activation promotes an alternatively-activated “M2” macrophage endotype, which supports tumorigenesis thorough suppression of T-cell mediated anti-tumor immunity, excessive STAT3 activity in epithelial cells and stimulation of tumor angiogenesis.

## References

[1] Poh AR (2015). Oncotarget.

[2] Poh AR (2017). Cancer Cell.

[3] Goldman A (2015). Nat Commun.

[4] Lowell CA (1994). Genes Dev.

[5] Ernst M (2002). J Exp Med.

[6] Qian BZ (2010). Cell.

[7] Xiao W (2008). J Clin Invest.

[8] Hong H (2007). Blood.

